# In Tube Integrated Electronic Nose System on a Flexible Polymer Substrate

**DOI:** 10.3390/s121013681

**Published:** 2012-10-12

**Authors:** Thomas Kinkeldei, Christoph Zysset, Niko Münzenrieder, Luisa Petti, Gerhard Tröster

**Affiliations:** Wearable Computing Lab, ETH Zurich, Gloriastarsse 35, 8092 Zurich, Switzerland; E-Mails: zysset@ife.ee.ethz.ch (C.Z.); muenzenrieder@ife.ee.ethz.ch (N.M.); petti@ife.ee.ethz.ch (L.P.); troester@ife.ee.ethz.ch (G.T.)

**Keywords:** flexible electronics, gas sensing, conductive polymer composite

## Abstract

The fabrication of electronic devices, such as gas sensors on flexible polymer substrates, enables the use of electronics in applications where conventional devices on stiff substrates could not be used. We demonstrate the development of a new intra-tube electronic-nose (e-nose) gas sensor device with multiple sensors fabricated and integrated on a flexible substrate. For this purpose, we developed a new method of fabricating a sensor array of four gas sensors on a flexible polymer substrate. The method allowed the use of lithography techniques to pattern different polymers with a broad range of solubility parameters. Conductive polymer composites were used as a gas sensitive layer due to the high stretchability of the material. Each of the 30 e-nose devices on one substrate was designed to fit on a polymer strip with a width of 2 mm. A single e-nose strip was successfully integrated into the inlet tube of a gas-measurement apparatus with an inner-tube diameter of 3 mm. Using the e-nose, we were able to differentiate between four different volatile solvent vapors. The tube-integrated e-nose outperformed a chamber-integrated e-nose of the same type in terms of response time and flow-rate influences. The sensor array inside the tube showed a faster response time and detected short pulses of analyte exposure compared to the same sensor array outside of the tube. We measured gas flow rates from 1,000 to 30 sccm without significant changes in sensor performance using this intra-tube e-nose prototype. The tube could be bent to radii <15 mm with a sensor performance similar to an unbent sensor.

## 1. Introduction

The display and photovoltaic industries have shown that fabricating electronics on flexible substrates composed of polymeric materials can compete or even outperform silicon devices. Flexible substrates make electronics usable in new fields of applications, as such devices on flexible substrates are bendable and applicable to curved surfaces with radii <10 mm. Compared to silicon substrates, flexible substrates are light weight and can be installed unobtrusively onto objects in our surrounding environment [1].

Flexible substrates offer the possibility of operating e-nose devices in spatially-constrained locations where it would be impossible to install rigid sensors on silicon or glass. A new flexible sensor in the medical field allows integration of a glucose and temperature sensor into a micro-catheter with a diameter <1 mm, enabling chemical sensing inside the human body [2]. In another example, a flow sensor based on a meander resistor, integrated into a tube with a diameter of 3 mm, allows gas flow monitoring inside a tube [3]. An inside-tube sensor outperforms sensors mounted on the outside of the tube and reacts faster due to the time delay for heat transport through the side walls of the tube. A giant magneto resistance (GMR) sensor attached to the outside of a flexible tube is capable of detecting magnetic objects that pass the sensor inside the tube [4]. A carbon nanotube gas sensor was placed into a glas tube with a diameter of 5 mm, enabling the detection of dimethyl methylphosphonate vapors [5]. Compared to a flat sensor the resistance response of the tube sensor slightly decreases by 5.6%. In a similar way, gas sensing with arrays could benefit from sensor-integration directly into tubing.

To detect the quality of supply air or to monitor exhaust air gas sensor arrays are installed in rooms or chambers. The time of flight for an analyte to interact with the gas sensor can be long, or in case of flow inside a chamber, might even not occur. The benefit of sensor placement directly into tubing is that supply or exhaust air will be transported through the tube and interact with an integrated sensor array without bypassing it due to air-flow dynamics within larger chamber spaces.

Materials for flexible substrate gas sensor integration have to be light weight, small and survive strain introduced by bending. Organic sensor materials outperform inorganic materials due to their ductility. Devices made out of conductive polymers (CP) filled with carbon black (CB) have shown to be stretchable above 50% strain without losing their functionality [6].

Carbon black polymer gas sensors are chemiresistors and change their resistance due to swelling upon exposure to an analyte [7]. Such sensors are used to measure relative humidity [8], vapors of solvents with concentrations <10 ppm [9] or gases like CO_2_ [8]. The combination of several CB sensors made of different polymer materials can be used as sensor array, a so-called electronic nose (e-nose), that is used to distinguish between gas mixtures [10].

Existing e-noses on flexible substrates are made of carbon nanotubes [11] used to differentiate between solvent vapours, or metal oxide nanoparticles [12] to detect toxic gases, but also of conductive polymers [13,14]. Gas sensor arrays of conductive polymers are used to detect substances in fires [13] or volatile solvent vapors in air [14].

Inorganic layers such as metal oxides have the benefit of detecting analytes in the range of several parts per billion (ppb) but are more influenced by bending strain and crack at a strain of about 2% [15]. Metal oxide gas sensors further need heating of the sensor to temperatures of about 200 – 300 °C. Compared with metal oxides, conductive polymer gas sensors show a reduced sensitivity but work at room temperature and survive strains above 2%.

Fabrication of CB/polymer e-noses is achieved by drop coating polymers onto several electrodes, on silicon [9] or on flexible substrates [14]. To fabricate devices <1 mm for the integration into tubes, micro-fabrication methods with lithography must be used. A significant problem arises from the solubility of the different CB/polymers in most of the standard lithography chemicals. This makes CB/polymer gas sensor fabrication incompatible when using standard lithography.

A new method of fabricating flexible and organic electronics is to use orthogonal lithography [16] that can be integrated on top of commonly-used CB/polymer gas sensors. By using dry etching, polymers can be structured with feature sizes <0.1 mm and sensor arrays for the integration into tubes can be achieved.

The objective of this study was to test a new method for the fabrication of a gas sensor prototype array on flexible polymer substrates using orthogonal lithography. The aim was to pattern several conductive polymer composite gas sensors that are designed to fit on a strip and successfully integrate into polymer tubing of a gas flow measurement apparatus with an inner diameter of 3 mm.

## 2. Materials and Methods

### 2.1. Sensor Fabrication

Four non-conductive polymers were used: polyisoprene (PIS), polystyrene (PS), poly(Nvinylpyrrolidone) (PVP), polyvinylbutyral (PVB) (Scientific Polymer Products). The solvents tetrahydrofuran (THF), toluene, acetone, methanol and isopropanol (IPA) were obtained from Sigma Aldrich. As conductive filler, Ensaco G350 from Timcal was used. Details for the polymer preparation can be found elsewhere [17]. Polyetherimide (PEI) foil (Ajedium Films) was used as flexible substrate material. The substrate had a thickness of 50 μm and a surface area of 76 mm × 76 mm.

**Figure 1.**
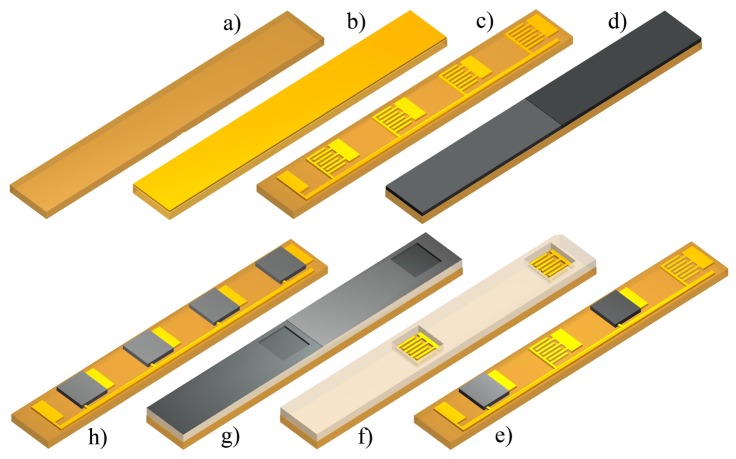
Fabrication process for the electronic nose using orthogonal resist: (**a**) cleaning of the substrate; (**b**) e-beam evaporation of gold; (**c**) patterned sensor structure; (**d**) gas sensitive layer spin-coating; (**e**) dry etched gas sensitive layer; (**f**) patterned fluorinated resist; (**g**) gas sensitive layer spin-coating; (**h**) lift-off patterned sensitive layer.

The fabrication scheme is depicted in Figure 1. The e-nose strip represents one of thirty e-nose strips that were processed on a single substrate in one fabrication run. PEI substrates were cleaned with Acetone/IPA and preshrunk in an oven at 190 °C for one day. For electrodes and contacts we deposited Ti/Au with a thickness of 5/100 nm using a Plassys e-beam evaporator Figure 1(b) and patterned it using a standard lift-off technique. The pattern for the sensor design consisted of an array of four interdigitated finger electrodes representing the gas sensors Figure 1(c). The width of the fingers as well as the gaps were 40 μm.

The sensors were designed to fit on a strip with a width of 2 mm and a length of 50 mm. The e-nose comprised 4 sensors, and 5 electrical contacts were necessary to connect each sensor individually. A common ground connected to all sensors and for each individual sensor element one contact.

The four CP sensing films (polyisoprene, polystyrene, poly(N-vinylpyrrolidone), polyvinylbutyral + CB) were deposited by spin-coating and patterned with a lift-off or dry etching technique. To be able to structure all four polymers using lithography, a fluorine based photoresist (OSCoR, Orthogonal Inc) was used. The PIS and PS sensors were patterned using a dry etching technique. On each half of the substrate we spin-coated the two different polymer solutions (Figure 1(d)). On top of the spin-coated films the fluorine photoresist was patterned as etching mask. An Oxford Plasmalab RIE system was subsequently used to dry etch the exposed gas sensing layers running a O_2_ plasma at 200 W, 700 μ̄ and 70 standard cubic centimeters per minute (sccm) flow for 5 min. Remaining resist was stripped from the substrate followed by cleaning in DI water (Figure 1(e)). A second fluorine resist layer was patterned as lift-off mask (Figure 1(f)). Again on each half of the substrate two different polymer solutions were spin-coated (Figure 1(g)). The resist was removed with the help of ultrasound, leaving the patterned polymer on the substrate (Figure 1(g)).

**Figure 2.**
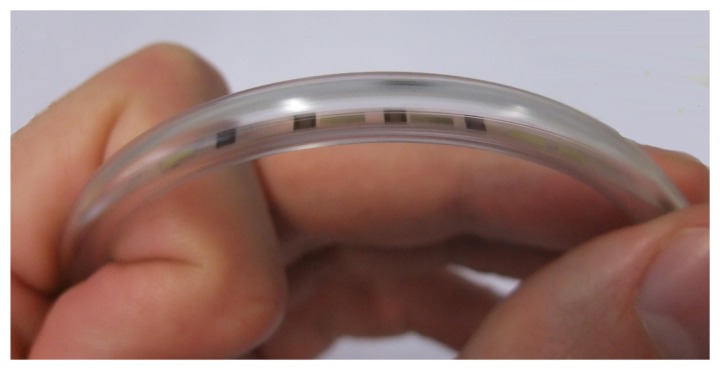
Photograph of the fabricated e-nose on a flexible substrate inserted into a polymer tube.

After fabrication, individual sensor strips were separated by cutting the substrate into 2-mm-wide strips using a Disco DAD 321 wafer saw with a 35 μm-wide blade-type 27HFDD. The strips were connected via 40 μm thin isolated copper wires. For a stable contact, the wires were glued to the contact pads using Epo-Tek H20E. The e-nose was inserted into a PET tube that had an inner diameter of 3 mm and served as gas inlet for a gas measurement chamber. A picture of the e-nose in the tube is shown in Figure 2.

### 2.2. Sensor Testing

Resistance measurements were done by connecting the wires to a multi-switch measure unit (Agilent 34980A). In an exsiccator (2 L volume), sensor measurements to distinguish different solvents were conducted at room temperature. The exsiccator was flushed with 1,000 sccm of synthetic air (80% N_2_ 5.0 and 20% O_2_ 5.0 with a humidity of 0%RH) using a Voegtlin mass flow controller. The test solvents were isopropanol, acetone, toluene and methanol. For each exposure we bubbled 40 sccm of the total flow through a bottle containing 40 mL of solvent and measured its response for 10 min. After the analyte, we exposed the sensor for 10 min to pure synthetic air to recover to the baseline resistance. The procedure was repeated for all solvents. Calibration measurements with acetone were done using a bottled gas standard containing a calibrated mixture of synthetic air plus 5,000 ppm acetone.

## 3. Results

### 3.1. E-nose Testing

**Figure 3.**
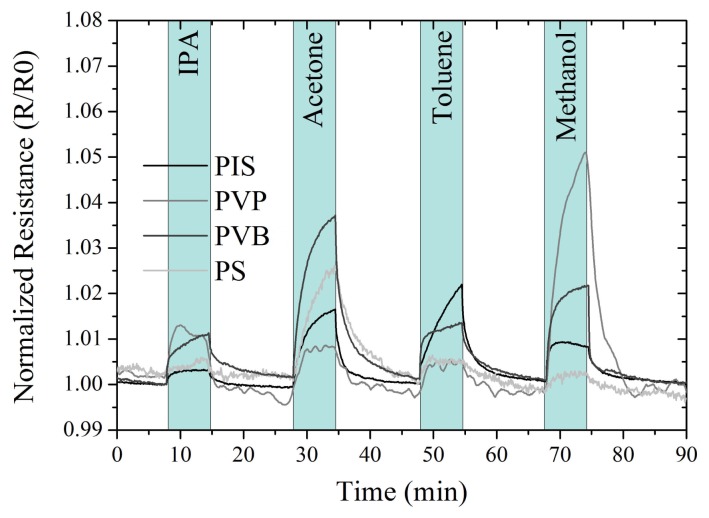
Normalized Resistance *vs*. time and exposure to solvents for the four different polymer gas sensors (PIS, PS, PVP, PVB). The volatile solvent vapors are isopropanol, acetone, toluene and methanol.

An intra-tube integrated e-nose strip was exposed to four different volatile solvent vapors (isopropanol, acetone, toluene and methanol). In Figure 3, the normalized resistance of each sensor *versus* time and exposure to solvents is plotted.

The sensors showed a repeated resistance increase upon the exposure to a volatile solvent vapor. After the solvent exposure, the sensors returned to their original baseline values. The sensor showed a characteristic resistance response for each combination of sensor element/solvent when exposed to the four volatile solvent vapors. The PVP sensor had a signal to noise ratio lower than the other sensors with peaks up to 0.2 percent of the resistance.

**Figure 4.**
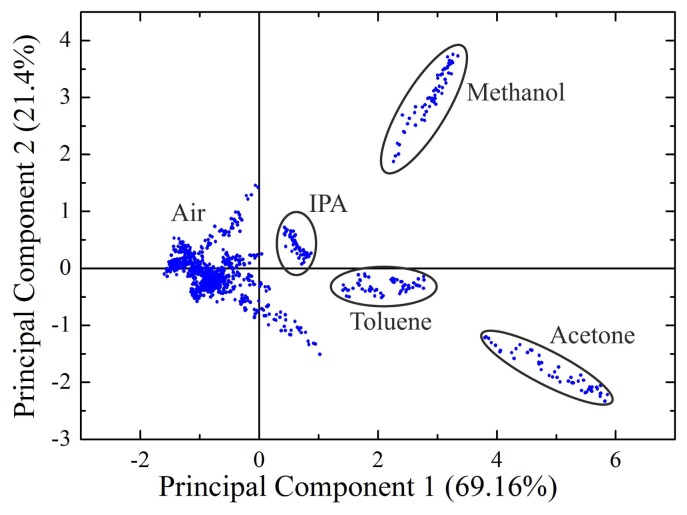
Principal component analysis of the sensor exposure to four different analytes: isopropanol, acetone, toluene and methanol. Each point in the plot represents a single measurement fragment of 1 s. The transition between a stable concentration of solvent or air is excluded from the plot.

Using the data from Figure 3, a principal component analysis (PCA) was performed to structure and visualize the data. For better visualization, we cut the sensor data by removing the transition phase from solvent to air and from air to solvent. Each measurement point in the PCA represents a 1 s measurement fragment of the original data. Figure 4 depicts the principal component analysis. The exposure to four volatile solvent vapors could be separated from each other using two principal components.

**Figure 5.**
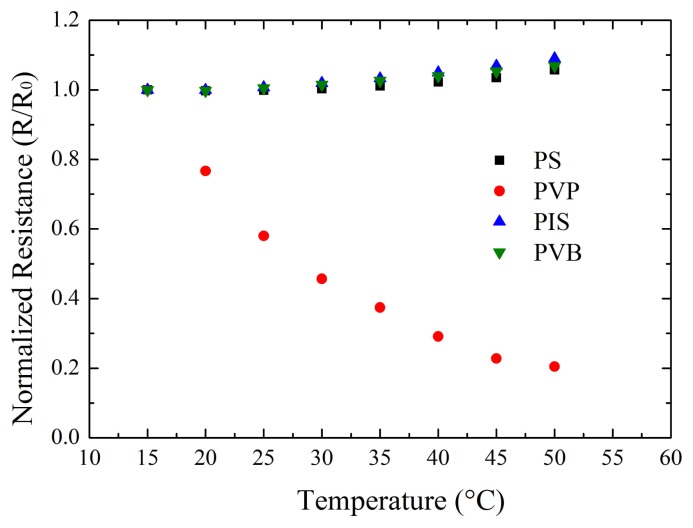
Normalized resistance *versus* temperature for the four different polymer gas sensors (PIS, PS, PVP, PVB).

An e-nose strip was placed into a temperature and humidity controlled chamber to test the influence of temperature on the e-nose at constant humidity and increasing temperature. The measurement was carried out at 50% RH and a temperature increase from 15 to 50 °C in steps of 5 °C. In Figure 5 the normalized resistance *versus* temperature for all sensors of the e-nose strip is shown.

The resistance increase for polyisoprene, polystyrene, and polyvinylbutyral showed a similar slope with a resistance increase of about 0.06% per 1 °C in the temperature range of 15–30 °C. Poly(N-vinylpyrrolidone) showed a considerable decrease of resistance with increasing temperature with resistance changes of more than 4% per 1 °C. The resistance of poly(N-vinylpyrrolidone) increases with water uptake of the polymer. The reason for the decrease could be due to a release of water from the polymer with increasing temperature [18].

### 3.2. Tube vs. Chamber

**Figure 6.**
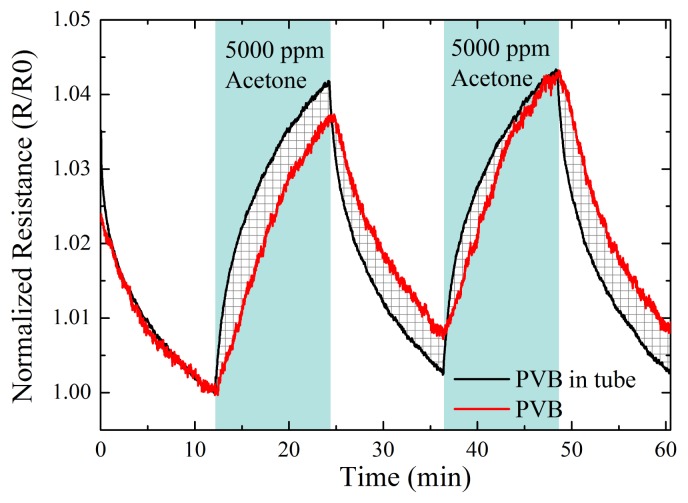
Normalized resistance over time and exposure to 5000 ppm acetone for a PVB gas sensors, showing the difference in reaction time. One PVB sensor is mounted inside the chamber and another inside the inlet tube of the chamber.

To test the reaction time of a sensor integrated into the tube *vs*. a sensor integrated into the chamber (the sensor is mounted 4 cm below the inlet), we measured the sensor response to repeated cycles of acetone exposure (5,000 ppm) and dry air. The sampling frequency of the measurement setup was 1 Hz. In Figure 6, the normalized sensor resistance of a PVB sensor *vs*. time is shown. The red curve represents the chamber integrated sensor and the black curve the tube integrated sensor.

The reaction time of the tube integrated sensor was faster than the chamber integrated sensor. The gas exposure duration for the chamber integrated sensor was not sufficient to establish a full sensor response. Further, the flushing duration was not sufficient to reach the steady baseline value after the first exposure to acetone.

### 3.3. Flow Measurement

**Figure 7.**
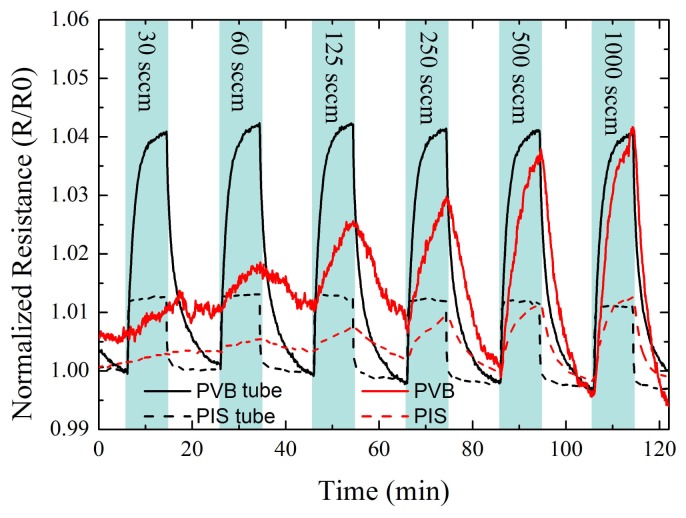
Normalized resistance over time and exposure to 5,000 ppm acetone with increasing flow rate. Two different sensor polymer materials are shown, *i.e.*, PIS and PVB. The flow rate was increased from 30 sccm to 1,000 sccm.

To determine the influence of the flow rate on the gas sensor response, measurements with an increasing flow rate at a constant volatile solvent vapor concentration were done. At each flow rate, the concentration of acetone in dry air was 5,000 ppm. In Figure 7 the normalized resistance of four different sensors *versus* time and exposure to solvents is shown. After each exposure cycle (exposure to solvent and subsequently dry air) the flow rate was increased with a total of 6 steps reaching from 30 sccm to 1,000 sccm.

The normalized resistance of the tube integrated sensors showed the same response for all flow rates. No decrease of the resistance amplitude to repeated exposure was detected even at the lowest flow rate of 30 sccm. The same sensors inside the chamber showed a continuous decrease of the resistance amplitudes with decreasing flow rates. A sensor inside the chamber showed a similar response only for a flow rate above 500 sccm compared with the tube integrated sensor. At a low flow rate of 30 sccm, no resistance change due to acetone exposure was measured. Starting at 60 sccm, the sensor showed a similar resistance increase and decrease to the volatile solvent vapor and the subsequent air exposure. With increasing flow rates, the sensor amplitudes became more dominant, reaching the same sensor response as the tube sensor at a flow rate of 500 sccm.

### 3.4. Tube Bending

**Figure 8.**
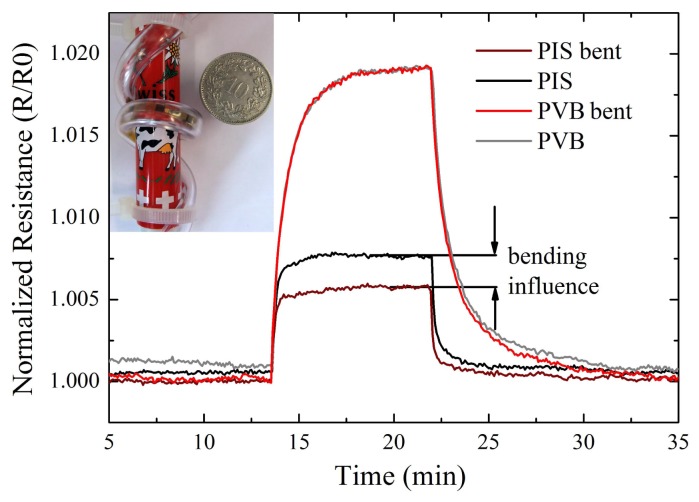
Normalized resistance over time and exposure to 2,500 ppm acetone. Shown are the same PIS and PVB sensors in a flat tube and in a tube bent to about 15 mm. The inset shows the bent tube with an integrated sensor wrapped around a pen.

To determine changes of the sensor due to tube bending, we compared the sensor response to acetone exposure in a flat tube and in a bent tube. Bending was achieved by wrapping the tube around a pen with a diameter of 15 mm (shown in the inset of Figure 8). The measurement in Figure 8 shows the normalized resistance over time and an exposure to acetone of 2,500 ppm. The sensitive layer of the shown sensors were PVB and PIS in a flat and bent tube.

From the plot it can be seen that the response of the sensor was dependent on the applied bending radius and the sensing material. PVB showed no significant influence in the bent state and in the flat state, while the PIS sensor showed a decreased response in the bent state. The decrease of sensor response for PIS was a change of about 0.2% at a total signal response of 0.75%. This corresponded to a decreased signal change of 26% at the same analyte exposure and concentration.

## 4. Discussion

### 4.1. Fabrication

To detect volatile solvent vapors with a broad range of solubility parameters, gas sensitive polymer films have to be chosen that match the same solubility parameter range. Corresponding to the solubility parameters of the polymers, solvents to dissolve the polymers for the fabrication have to be selected. This creates challenges with lift-off patterning using conventional photoresists. This is because conventional photoresists are solved by THF and acetone, two solvents used to dissolve the polymers for the presented electronic nose. Patterning with dry etching creates challenges for conventional photoresist, due to the used developers and strippers for patterning micro-structures. The developers consist of water diluted bases that are compatible with the used polymers PIS, PVB and PS but dissolves PVP. The stripper solvents, for example NMP or acetone, are compatible with PVP and PIS but dissolve PS and PVB. This implies restrictions for the used strippers or developers that cannot be met with conventional photoresists.

Lithography with fluorinated resists, the so-called orthogonal lithography, uses a special developer and stripper based on fluorinated carbon compounds that are benign to non-fluorinated polymers, thus making orthogonal lithography compatible with the used polymers for the e-nose. One problem arising with the use of the fluorinated resist is the solubility in THF. For this reason, PS and PIS (which are dissolved in THF) could not be fabricated using a lift-off method but had to be structured using a dry etching method.

**Figure 9.**
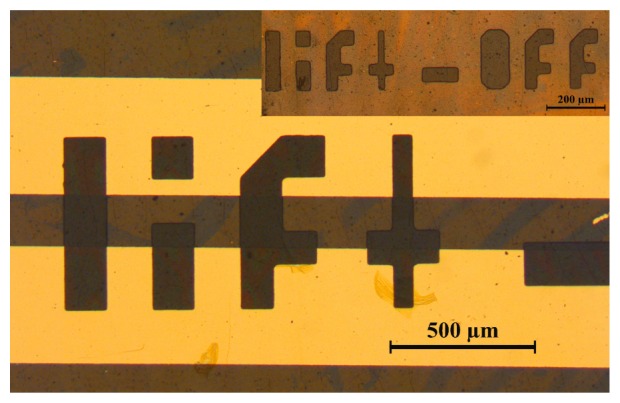
Micrograph of a structured gas sensor layer onto metal electrodes on a PI substrate. Structuring was achieved with dry etching. The inset shows a patterned polymer layer on a PI substrate with feature sizes below 50 μm.

With the use of fluorinated resist, polymer structures of 50 μm were fabricated and the resulting miniaturized sensor systems on flexible polymer substrates were made. An example of such structured polymers is shown in Figure 9.

### 4.2. Intra-tube vs. Chamber E-nose

Using flexible substrates, the integration of a complete e-nose into tubing of a gas-measurement apparatus was possible. The tube containing the e-nose was still fully flexible and could be used as chamber inlet of the gas-measurement apparatus. Bending of the inlet tubing was possible to a radius of 15 mm before tube deformation such as folds or buckles occurred. The e-nose strip adapted to the applied bending radii. Due to bending inside the tube, the sensor resistances changed with maximum changes below 5%. The maximal possible bending diameter of 15 mm corresponded to a calculated strain (according to [15]) of about 0.4%. This value was still in the safe regime of bending where only elastic deformation occurs. Critical bending starts at tensile bending strain of about 1% [15]. Measurements with exposure to analytes showed that the sensitivity is partly altered by bending the e-nose. PVB did not show any change of performance during the exposure to solvents, while PIS showed a decreased performance with a decrease in signal amplitude of 26%. This difference might be attributed to a difference of the local bending radius of parts of the strip inside the tube. The different bending radii lead to a changing ability of the e-nose to differentiate between solvents. To overcome this problem, single gas sensors have to be fabricated within closer distance to achieve a similar bending radius for both sensors during bending. Having similar bending radii, each sensor then shows a similar resistance change [17]. This information can be used to differentiate resistance changes due to bending with those due to gas exposure.

An advantage of the intra-tube e-nose was the faster reaction time. This benefit was achieved due to the low volume inside the tube where the sensor was installed. As one would expect from standard gas kinematics, in the tube there is no dilution of the introduced gases with the air inside the chamber. Hence the time for adsorption and the resulting swelling of the sensitive material are the limiting factors for the sensor reaction, whereas the time for establishing a stable concentration inside the chamber is not. Along with the faster reaction time, the recovery time—from the full sensor response upon exposure back to the baseline resistance—also decreased. In Figure 6, for the second analyte exposure cycle, the chamber integrated e-nose could not return to the base line resistance after the exposure to acetone. This showed that the adsorption and swelling is faster than the desorption and relaxing of the material. From the experiment with increasing flow rates in Figure 7, it can also be seen that depending on what type of polymer is used, the reaction time differs. PIS reacted nearly immediately to the exposure of the solvent, while PVB has a delay in reaction time.

A further advantage of the intra-tube e-nose was the independence on the flow rate, again related to the dilution of the introduced gases and according to standard gas kinematics. A small flow rate will take a larger amount of time until the concentration inside the chamber is stabilized or that the local space around the sensor below the inlet (the distance of inlet and sensor was 4 cm) has reached a certain concentration. With the tube integrated sensor it was also possible to detect small traces of leakage inside the tubing. Short pulses of solvent exposure of below 5 seconds could be detected even at flow rates of 30 sccm, which was not detected by the chamber integrated sensor.

## 5. Conclusions

Substrate flexibility, large area manufacturing, unobtrusive installation and improved resilience against bending strain are key advantages of electronics on flexible substrates. In this paper we make use of these properties by integrating an electronic nose system into the flexible inlet tubing of a gas-measurement apparatus.

The e-nose consisted of four different polymer composite gas sensors on a flexible polymer foil. Due to solubility conflicts between common photoresists and the used gas sensitive polymer solutions, orthogonal lithography, based on fluorinated polymer resists, was used. It was possible to achieve feature sizes of the spin-coated gas sensitive layers <50 μm. Introducing orthogonal lithography to structure polymer layers of an e-nose, we achieved a reduced integration density of multiple sensors on flexible substrates compared with the state-of-the-art flexible sensors [14]. The reduced integration density as well as the bendability allowed the installation of an e-nose inside the inlet-tube (inner dimension of 3 mm) of a gas measurement setup.

The intra-tube integrated e-nose showed an improved behavior in terms of reaction time and flow rate compared with a similar e-nose inside the chamber of the gas-measurement apparatus. The intra-tube integrated e-nose could further adapt to the curvature change of the used tubing and detect volatile solvent vapors in the flat and in the bent state. Depending on the polymer, a change in sensor response before and after bending was observed.

The present approach is a first step to demonstrate the versatile use of gas sensor arrays or in general of electronics on flexible substrates and the improvement of existing sensor solutions. Existing hurdles for electronics to be installed in point of need are often spatially-constrained locations, but also include the change of environmental properties (such as thermal mass, friction, weight) or even acceptance by aesthetic reasons. Compared with bulky and rigid sensors on Si [9], these hurdles can be overcome with flexible electronics as they are thin, transparent, light weight, conformable and have a low thermal mass. These properties together can create new sensor solutions or improve existing technologies.
